# Characteristics and difference of respiratory diseases in Korean adults aged ≥40 years: A cross‐sectional study

**DOI:** 10.1111/crj.13558

**Published:** 2022-11-15

**Authors:** Yoon Jae Won, Sook‐Hyun Lee, Yu‐Cheol Lim, Yoon Jae Lee, Maurits Van den Noort, Beom‐Joon Lee, In‐Hyuk Ha

**Affiliations:** ^1^ Jaseng Hospital of Korean Medicine Seoul Republic of Korea; ^2^ Jaseng Spine and Joint Research Institute Jaseng Medical Foundation Seoul Republic of Korea; ^3^ Research Group of Pain and Neuroscience, WHO Collaborating Center for Traditional Medicine, East‐West Medical Research Institute Kyung Hee University Seoul Republic of Korea; ^4^ Department of Internal Korean Medicine Kyung Hee University Korean Medicine Hospital Seoul Republic of Korea; ^5^ Division of Allergy, Immune and Respiratory System, Department of Internal Medicine, College of Korean Medicine Kyung Hee University Seoul Republic of Korea

**Keywords:** asthma, COPD, KNHANES, Korean National Health and Nutrition Examination Survey

## Abstract

**Purpose:**

National big data pertaining to the status of common respiratory diseases is essential to devising appropriate policies to promote proper treatment and prevention of respiratory diseases amid the prolonged coronavirus disease 2019 (COVID‐19) pandemic. The aim of this study is to investigate the prevalence of common respiratory diseases and their association with sociodemographic characteristics, comorbidities, and medical history using 11 years (2008–2018) of the Korea National Health and Nutrition Examination Survey (KNHANES) data, ultimately to present foundational data for policy decision making and disease prevention measures.

**Methods:**

Among the participants of the KNHANES survey (2008–2018), 93 028 adults aged ≥40 years who underwent a lung function test were included in this cross‐sectional study. The participants were divided into four groups: Asthma, chronic obstructive pulmonary disease (COPD), asthma + COPD, and no respiratory disease. Their data were analyzed for demographic factors, health behavior, and disease‐related factors. Multiple logistic regression was used to calculate the odds ratio (OR) adjusted for sex, age, household income, educational level, occupation, body mass index (BMI), smoking status, alcohol consumption, physical activity, and comorbidities.

**Results:**

Of all participants, 1.83%, 12.63%, and 1.27% had only asthma, only COPD, and asthma + COPD, respectively. With respect to the patients with asthma who also had asthma + COPD, the OR of asthma + COPD was 5.272 in underweight patients and 6.479 in patients aged ≥70 years. Meanwhile, a high association between COPD and asthma + COPD was found in female patients, whereas asthma was more highly associated with asthma + COPD in male patients.

**Conclusion:**

The study confirmed that old age, sex, smoking status, BMI, previous history of atopic dermatitis, and lung cancer were independent risk factors for asthma, COPD, and asthma + COPD. The present study demonstrated the need for a multidisciplinary integrative approach to respiratory diseases, and the findings could be used for developing policies for the treatment of COVID‐19 and respiratory diseases and the prevention of infectious diseases.

## Graphical abstract

The odds ratio (OR) of having asthma + COPD among patients with asthma was lower in females and older people. Regarding BMI, risk of asthma + COPD was higher in underweight patients with asthma as compared with those with normal BMI, whereas the association was lower in overweight subjects. In contrast to asthma patients, females, older ages, and overweight patients with COPD had higher risk of asthma + COPD.

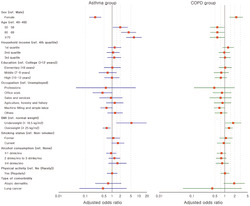

## 1 INTRODUCTION

The prevalence of respiratory diseases is gradually increasing due to worsening air pollution in recent years and the aging of society. Global Burden of Disease Study 2020 reported that deaths associated with lower respiratory tract infections have decreased over the past 20 years, and yet many deaths are still caused by respiratory diseases.^1^, ^2^

Asthma is a heterogeneous chronic inflammatory disease with several etiologies, and its symptoms include breathing difficulty and coughing.^3^ In 2015, asthma was suspected to be one of the most common chronic respiratory diseases, affecting 358 million people worldwide. The prevalence rate of asthma has been reported to range between 0.7% and 11.9% in Asian countries, including South Korea.^4^

Chronic obstructive pulmonary disease (COPD) is a lung disease characterized by irreversible airflow limitation that has become a public health issue with a high prevalence and mortality rate. According to the World Health Organization (WHO), COPD will become the third leading cause of death by 2030. The socio‐economic burden associated with COPD is substantial in Korea. The direct medical cost per COPD patient was $2800 in 2009, which was slightly higher than that of Canada and the United States.^5^ COPD has a direct correlation with smoking status, but in some countries, air pollution, occupational exposure, and indoor air pollution due to the use of biomass are also key causes.^6^

The definition of asthma + COPD remains controversial, with no commonly accepted definition. In clinical practice, diagnosis of asthma or COPD is suggested if three or more of the symptoms corresponding to asthma or COPD are present. On the other hand, diagnosis of asthma + COPD should be considered when the patient presents symptoms of asthma and COPD alike.^7^ In general, asthma + COPD is diagnosed when asthma features are accompanied by irreversible airflow limitation.^8^ asthma + COPD progresses faster and is associated with poorer health‐related quality of life, higher frequency of exacerbation, comorbidities, and health care utilization rate than asthma or COPD alone. The high economic burden of asthma + COPD, as compared with asthma and COPD, is well documented.^9^ However, recommendations for managing asthma + COPD are ambiguous and are inferred from guidelines for asthma or COPD alone.^10^

Previous studies have used the Korean National Health and Nutrition Examination Survey (KNHANES) data to investigate respiratory diseases. A study used data from the 2007–2012 KNHANES to divide patients with asthma + COPD into asthma‐dominant and COPD‐dominant groups to investigate their socio‐economic status and quality of life. The results showed that the asthma‐dominant asthma + COPD group had lower socio‐economic status and poorer quality of life than the COPD‐dominant asthma + COPD group.^11^ Another study used data from the 2007–2011 KNHANES to assess the risk factors for COPD among Korean nonsmokers. The results showed that older age, male sex, low body mass index (BMI), asthma, and tuberculosis were risk factors for COPD among nonsmokers.^12^

The COVID‐19 pandemic remains active since the index case in late 2019. COVID‐19 is known to have a greater repercussion on individuals with preexisting respiratory conditions, such as asthma and COPD.^13^ This study investigated the status of respiratory diseases in South Korea until 2018, before the advent of COVID‐19. This data can be used in subsequent studies to comparatively analyze the status of respiratory diseases prior to and after the advent of COVID‐19.

As described, typical respiratory diseases include asthma, COPD, and asthma + COPD, but each disease has various risk factors according to its own characteristics. Previous studies have focused on the associations of a single factor, but no study has investigated that specific factor, among the multitude of complex factors, which shows the strongest association. Moreover, while previous studies have investigated asthma, COPD, and asthma + COPD separately, and some have investigated two of these diseases, no study has compared all three diseases during the period between 2008 and 2018. Accordingly, the present study aims to analyze the risk factors that influence asthma, COPD, and asthma + COPD and compare the risk factors using data from Korean adults aged ≥40 years from the KNHANES 2008–2018.

## 2 METHODS

### 2.1 Study design

KNHANES is a nationwide survey conducted by the Korea Centers for Disease Control and Prevention (KCDC), and the present study used data from the fourth to seventh KNHANES survey (KNHANES IV‐VII). Starting from 1998, KNHANES has been conducted in 3‐year waves. It is a nationwide health and nutrition survey with representativeness and reliability that has been conducted annually since 2007. The survey collects information on the general characteristics, health behavior, chronic disease status, and nutritional intake status of the Korean population. The findings are used as basic data for establishing health care policies.

### 2.2 Study participants

**FIGURE 1:**
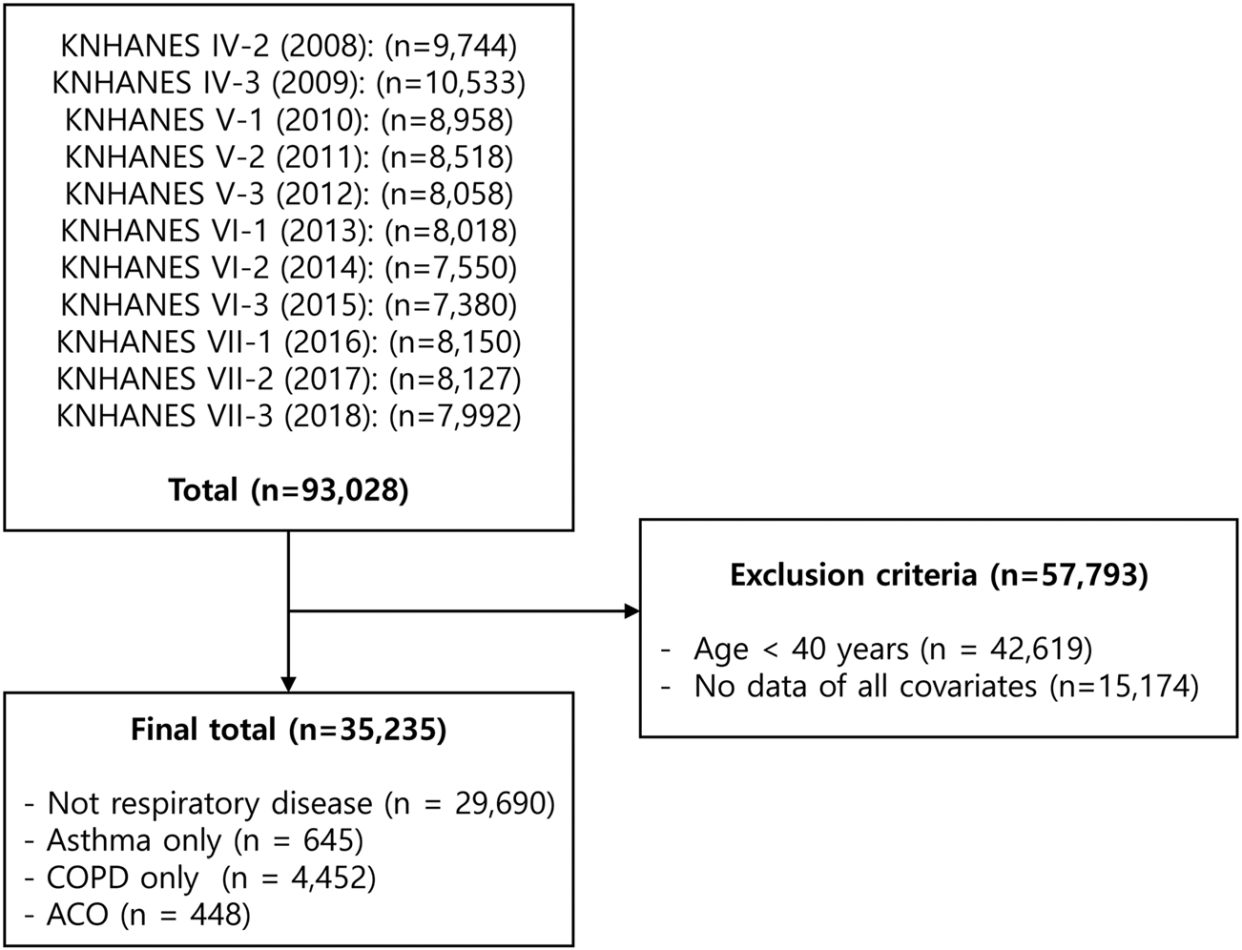
Flow diagram showing the number of participants who were excluded and the number of data that were analyzed. COPD, chronic obstructive pulmonary disease

The present study used data from the KNHANES IV‐VII, collected between January 2008 and December 2018. In the present study, 93 028 individuals were surveyed, of which 35 235 adults aged ≥40 years who took part in a lung function test were included in the final analysis. Of the 93 028 surveyed individuals, 42 619 individuals aged <40 years and 15 174 individuals with missing data regarding asthma and COPD were excluded. Consequently, the analysis of the present study included a total of 35 235 individuals (Figure 1).

### 2.3 Study tools

#### 2.3.1 General characteristics

Among the health questionnaire survey items in the KNHANES IV‐VII, the present study analyzed sex, age, household income, educational level, occupation, BMI, smoking status, alcohol consumption, physical activity, previous history, lung function test, and the Global Initiative for Chronic Obstructive Lung Disease (GOLD) criteria.

Sex, age, household income, educational level, and occupation were selected as demographic characteristics. Age was divided into 40–49, 50–59, 60–69, and ≥70 years, whereas the educational level was divided into elementary school graduate or lower, middle school graduate, high school graduate, and college graduate or higher for individuals aged ≥40 years. For household income, the household equivalent income quartiles from the KNHANES (“low,” “middle‐low,” “middle‐high,” and “high”) were used. The occupation was divided into unemployed; professional; office work; sales and services; agriculture, forestry, and fishery; machine fitting and simple labor; and others.

BMI, smoking status, alcohol consumption, physical activity, previous history, lung function test, and GOLD criteria were analyzed as health‐related characteristics. BMI was divided into underweight (BMI < 18.5), normal (18.5 ≤ BMI < 25), and overweight (25 ≥ BMI) based on anthropometric results to calculate body weight (kg)/height^2^ (m^2^), in accordance with the WHO standards.^12^ Smoking status was divided into current smoker, former smoker, and nonsmoker, whereas alcohol consumption was divided into none, ≤1, 2–3, and ≥4 drinks based on the monthly frequency of consumption. Physical activity was divided based on the frequency of walking during 1 week, with “No” for not walking at all and “Yes” for just once or two to seven times. Excluding asthma and COPD, we identified individuals who indicated a previous diagnosis of atopic dermatitis and lung cancer, which are typically associated with lung disease. Lung function test results were divided into four parameters: Predicted percentage of forced vital capacity [FVC (% pred)], predicted percentage of forced expiratory volume [FEV1 (% pred)], FEV1/FVC, and FEV1/FVC < 0.7. The four stages of the GOLD criteria were used as follows: stage 1 (mild) = FEV ≥ 80%; stage 2 (moderate) = 50% ≤ FEV < 80%; stage 3 (severe) = 30% ≤ FEV < 50%; and stage 4 (very severe) = FEV < 30%.^14^

#### 2.3.2 Definition of asthma group

The asthma group was defined as patients answering “Yes” to whether they had been diagnosed with asthma by a doctor.

#### 2.3.3 Definition of COPD group

The COPD group was defined based on the results of the lung function test. FVC (% pred; mean ± SE) and FEV1 (% pred; mean ± SE) were determined and used to calculate FEV1/FVC (mean ± SE). Patients with FEV1/FVC < 0.7 were defined as having COPD.^15^, ^16^

#### 2.3.4 Definition of asthma + COPD group

Individuals with both asthma and COPD were defined as the asthma + COPD group. Hence, this group was comprised of those who responded “Yes” to the question of whether they had been diagnosed with asthma by a doctor and who had an FEV1/FVC < 0.7.^17^

### 2.4 Statistical analysis

KNHANES is a nationwide sample survey that used complex sample analysis considering weights, stratification variables, and cluster variables. The distribution of the characteristics of the study participants was expressed as frequency and percentage, while continuous data were expressed as mean and 95% confidence interval (95% CI). To identify the risk factors for asthma + COPD among patients with respiratory diseases, we performed logistic regression analysis with asthma + COPD as a binary dependent variable. Additionally, the risk factors for each respiratory disease were also analyzed using each respiratory disease (Asthma, COPD, asthma + COPD) as the dependent variable. Sex, age, household income, education, occupation, BMI, smoking status, alcohol consumption, physical activity, and type of comorbidity were included as covariates in the logistic regression analysis. Furthermore, the annual status of asthma, COPD, and asthma + COPD from 2008 to 2018 was investigated. The logistic regression results were presented as odds ratio (OR) and 95% CI. The data used in the present study were statistically analyzed using SAS V9.4 (SAS Institute Inc, Cary, NC, USA) with the significance level set to <0.05 (two‐tailed).

## 3 RESULTS

Among all Korean adults aged ≥40 years who underwent a lung function test, 1.83%, 12.63%, and 1.27% had asthma, COPD, and asthma + COPD, respectively. In the asthma group, the prevalence was three times higher in women than in men, whereas the prevalence among men was 3.2 and 1.2 times higher than that in women in the COPD and asthma + COPD groups, respectively. Table 1 shows the basic characteristics of the participants. Among the demographic characteristics, the age brackets with the highest number of participants were 40–49 years (31.28%), 60–69 years (32.58%), and ≥70 years (34.86%) in the asthma, COPD, and asthma + COPD groups, respectively. Regarding household income, asthma showed the highest prevalence in the high‐income group (26.15%), whereas COPD and asthma + COPD showed the highest prevalence in the low‐income group (29.25% and 39.48%, respectively). Whereas the most common education level was high school in the NRD group (35.77%), that in each of the three respiratory disease groups was elementary school (Asthma: 36.81%, COPD: 37.45%, asthma + COPD: 49.99%). In all groups, unemployed individuals accounted for the greatest percentage, and most patients had normal weight. While many individuals in the NRD and asthma group were nonsmokers, the percentage of smokers was relatively high in the COPD groups. Similarly, many individuals in the NRD, asthma, and asthma + COPD groups were nondrinkers (NRD: 26.88%, asthma: 41.74%, asthma + COPD: 40.9%), whereas the COPD group showed a higher proportion of current drinkers (36.15%). Concerning physical activity, all the four groups showed a higher proportion of subjects who regularly participated in physical activity (NRD: 82.69%, asthma: 82.54%, COPD: 79.43%, asthma + COPD: 78.86%).

**TABLE 1:** Characteristics of Korean participants with asthma, COPD, and asthma + COPD in the KNHANES 2008–2018

Variables		NRD (*n* = 29 690)		Asthma only (*n* = 645)		COPD only (*n* = 4452)		Asthma + COPD (*n* = 448)	
		*n*	%	*n*	%	*n*	%	*n*	%
Sex^a^	Men	11 701	46.25	129	24.87	3300	76.43	241	55.61
	Women	17 989	53.75	516	75.13	1152	23.57	207	44.39
Age (y)^a^ ^,^ ^b^		53.78 (53.60–53.95)		56.58 (55.53–57.63)		62.85 (62.46–63.24)		63.46 (62.08–64.83)	
	40–49	9567	40.28	141	31.28	310	11.65	42	14.32
	50–59	9297	33.98	167	30.12	840	26.22	69	21.12
	60–69	6856	16.91	185	21.82	1584	32.58	143	29.69
	≥70	3970	8.83	152	16.78	1718	29.56	194	34.86
Household income^a^	1st quartile	5590	15.22	199	25.36	1502	29.25	191	39.48
	2nd quartile	7401	24.68	146	22.25	1226	26.90	108	22.95
	3rd quartile	7786	27.98	145	26.23	878	22.52	81	19.80
	4th quartile	8913	32.12	155	26.15	846	21.33	68	17.76
Education^a^	Elementary (<6 years)	8186	21.36	299	36.81	1876	37.45	235	49.99
	Middle (7–9 years)	4318	14.01	92	16.53	754	17.40	64	13.28
	High (10–12 years)	9654	35.77	141	24.00	1136	28.02	89	20.62
	College (>12 years)	7532	28.86	113	22.65	686	17.13	60	16.12
Occupation^a^	Unemployed	14 390	46.59	379	54.73	2507	54.94	291	63.73
	Professions	607	2.51	7	1.18	82	2.02	3	0.77
	Office work	2670	10.62	49	10.08	182	4.99	19	4.44
	Sales and services	2201	8.65	25	5.16	165	4.73	11	3.21
	Agriculture, forestry and fishery	2020	7.25	31	5.95	143	4.13	19	4.42
	Machine fitting and simple labor	4691	14.27	94	13.76	838	17.01	60	12.01
	Others	3111	10.10	60	9.14	535	12.18	45	11.41
BMI (kg/m^2^)^a^ ^,^ ^b^		24.32 (24.28–24.37)		25.02 (24.59–25.46)		23.71 (23.61–23.81)		23.91 (23.55–24.26)	
	Underweight	490	1.59	8	0.98	124	2.59	24	4.96
	Normal weight	18 002	60.08	342	55.06	3012	66.89	271	59.67
	Overweight	11 198	38.33	295	43.96	1316	30.52	153	35.37
Smoking status^a^	Nonsmoker	18 779	57.33	488	72.01	1381	29.13	199	44.30
	Former	4850	18.55	66	12.05	1422	31.81	116	25.09
	Current	6061	24.12	91	15.94	1649	39.06	133	30.61
Alcohol consumption^a^	None	9211	26.88	297	41.74	1442	29.29	199	40.90
	≤1 drink/mo	8460	27.61	188	28.32	858	19.15	93	19.77
	2 drinks/mo to 3 drinks/mo	9821	37.13	137	25.61	1488	36.15	110	27.77
	≥4 drinks/mo	2198	8.39	23	4.33	664	15.40	46	11.56
Physical activity (Regular Walking)^a^	No (Rarely)	5203	17.31	119	17.46	903	20.57	98	21.14
	Yes (Regularly)	24 487	82.69	526	82.54	3549	79.43	350	78.86
Type of comorbidity ^a^	Atopic dermatitis	415	1.40	21	4.31	48	1.45	13	2.84
	Lung cancer	25	0.07	1	0.16	22	0.43	1	0.13
Lung function^b^	FVC (% pred)	92.01 (91.82–92.19)		89.18 (87.96–90.40)		90.29 (89.78–90.80)		84.12 (82.27–85.98)	
	FEV1 (% pred)	92.88 (92.70–93.06)		89.05 (87.87–90.24)		78.11 (77.60–78.62)		65.76 (63.64–67.88)	
	FEV1/FVC	0.80 (0.80–0.80)		0.78 (0.78–0.79)		0.64 (0.64–0.64)		0.58 ± (0.57–0.59)	
GOLD	Stage 1	26 011	87.50	498	78.64	2107	47.04	106	23.92
	Stage 2	3655	12.43	145	20.89	2162	48.74	247	55.06
	Stage 3	24	0.07	2	0.47	162	3.69	83	18.85
	Stage 4	‐		‐		21	0.53	12	2.17

^a^ Categorical variables are presented as frequencies and percentages;

^b^ Continuous variables are presented means and 95% confidence intervals.

Abbreviations: BMI, body mass index; COPD, chronic obstructive pulmonary disease; FEV, forced expiratory volume.

Among the disease‐related factors, the lung function test results showed a decreasing mean FEV1/FVC value in the order of the NRD (0.80), asthma (0.78), COPD (0.64), and asthma +COPD (0.58) groups. With respect to the GOLD criteria, the NRD and asthma groups showed the highest prevalence in stage 1; the COPD group, in stages 1 and 2; and the asthma + COPD group, in stage 2.

Table S1 shows the logistic regression analysis results on the association between asthma and asthma + COPD, as well as the association between COPD and asthma + COPD. The results were adjusted for all demographic factors, health behavior, and disease‐related factors. The OR of having asthma + COPD among females with asthma, as compared with males with asthma, was 0.234 (95% CI 0.140–0.392), indicating a significantly negative association of asthma + COPD among females with asthma. With respect to age, the association between asthma and asthma + COPD increased with increasing age compared with those aged 40–49 years. In particular, the OR of having asthma + COPD among those aged ≥70 years was 6.479 (95% CI 3.327–12.615) in the asthma group, which showed the highest association. Regarding BMI, the OR of having asthma + COPD was high, with a value of 5.272 (95% CI 1.444–19.246) in underweight patients with asthma as compared with those with normal BMI, while the association was lower in overweight subjects (OR: 0.627; 95% CI 0.445–0.884). The OR of having asthma + COPD among females with COPD, as compared with males with COPD, was 2.235 (95% CI 1.397–3.577), indicating a significant association between asthma + COPD and females with COPD. In contrast to asthma, the association with asthma + COPD appeared higher among women with COPD than among men with COPD. With respect to age, the association between COPD and asthma + COPD decreased with increasing age as compared with those aged 40–49. Concerning BMI, the OR of having asthma + COPD in patients with COPD who were underweight was 1.804 (95% CI 1.024–3.177), whereas patients who were overweight were thought to have a higher risk of asthma + COPD (OR: 1.348, 95% CI 1.035–1.757).

Table 2 shows the results of the logistic regression analysis (adjusted for all demographic factors, health behavior, and disease‐related factors) on the associations among the asthma, COPD, and asthma + COPD groups.

**TABLE 2:** Association of risk factors for asthma, COPD, and asthma + COPD among Korean participants in KNHANES 2008–2018

		Asthma			COPD			Asthma + COPD		
Category		OR^a^	95% CI		OR^a^	95% CI		OR^a^	95% CI	
Sex	Men (ref.)	1.000			1.000			1.000		
	Women	2.550	1.764	3.687	0.294	0.255	0.338	0.652	0.411	1.033
Age (y)	40–49 (ref.)	1.000			1.000			1.000		
	50–59	0.972	0.713	1.325	2.592	2.205	3.046	1.566	0.983	2.495
	60–69	1.117	0.796	1.568	6.676	5.678	7.848	3.720	2.304	6.008
	≥70	1.274	0.878	1.849	12.327	10.383	14.634	6.399	3.824	10.709
Household income	1st quartile	1.191	0.867	1.638	1.222	1.062	1.405	1.568	1.040	2.366
	2nd quartile	0.885	0.658	1.192	1.053	0.920	1.206	0.994	0.677	1.461
	3rd quartile	1.078	0.798	1.455	1.019	0.889	1.167	1.008	0.685	1.483
	4th quartile (ref.)	1.000			1.000			1.000		
Education	Elementary (<6 years)	1.482	1.043	2.106	1.478	1.267	1.723	1.533	0.971	2.419
	Middle (7–9 years)	1.368	0.946	1.978	1.303	1.107	1.534	0.970	0.590	1.593
	High (10–12 years)	0.836	0.614	1.139	1.166	1.012	1.343	0.842	0.552	1.282
	College (>12 years) (ref.)	1.000			1.000			1.000		
Occupation	Unemployed (ref.)	1.000			1.000			1.000		
	Professions	0.872	0.348	2.186	0.803	0.599	1.077	0.350	0.078	1.559
	Office work	1.305	0.881	1.933	0.898	0.725	1.112	0.702	0.377	1.305
	Sales and services	0.897	0.555	1.452	0.956	0.776	1.180	0.651	0.296	1.430
	Agriculture, forestry and fishery	0.753	0.487	1.163	1.170	0.928	1.475	0.977	0.560	1.702
	Machine fitting and simple labor	0.929	0.687	1.255	1.060	0.938	1.198	0.684	0.472	0.991
	Others	0.719	0.505	1.024	1.148	1.005	1.312	0.890	0.583	1.359
BMI (kg/m^2^)	Normal weight (ref.)	1.000			1.000			1.000		
	Underweight	0.598	0.240	1.492	1.405	1.042	1.896	2.463	1.426	4.253
	Overweight	1.255	1.038	1.518	0.693	0.633	0.759	0.870	0.681	1.112
Smoking status	Nonsmoker (ref.)	1.000			1.000			1.000		
	Former	1.261	0.828	1.923	1.546	1.336	1.790	1.728	1.095	2.726
	Current	1.272	0.883	1.830	2.336	2.029	2.689	2.145	1.320	3.484
Drinking status	None (ref.)	1.000			1.000			1.000		
	≤1 drink/mo	0.779	0.615	0.987	0.885	0.786	0.996	0.721	0.525	0.990
	2 drinks/mo to 3 drinks/mo	0.699	0.532	0.918	0.884	0.788	0.992	0.708	0.497	1.007
	≥4 drinks/mo	0.578	0.339	0.987	0.919	0.795	1.064	0.793	0.507	1.242
Physical activity (regular walking)	No (rarely) (ref.)	1.000			1.000			1.000		
	Yes (regularly)	1.085	0.840	1.402	0.958	0.859	1.069	0.991	0.742	1.324
Type of comorbidity	Atopic dermatitis	3.175	1.792	5.624	1.059	0.714	1.569	2.093	1.050	4.171
	Lung cancer	2.115	0.272	16.432	3.122	1.650	5.907	1.188	0.161	8.771

^a^ Odds ratios with adjustments using logistic regression models adjusted for sex, age, household income, education, occupation, BMI, smoking status, alcohol consumption, physical activity, and types of comorbidity.

Abbreviations: BMI, body mass index; CI, confidence interval; COPD, chronic obstructive pulmonary disease; KNHANES, Korean National Health and Nutrition Examination Survey; aOR, adjusted odds ratio.

Among the demographic factors, the female sex in the asthma group showed a higher association than the male sex, with an OR of 2.550 (95% CI 1.764–3.687), whereas for females in the COPD group, the association was significantly lower, with an OR of having asthma of 0.294 (95% CI 0.255–0.338). In the asthma group, there was no significant difference according to age. In the COPD group, the OR of having COPD increased with age: 50–59 years (2.592, 95% CI 2.205–3.046), 60–69 years (6.676, 95% CI 5.678–7.848), and ≥70 years (12.327, 95% CI 10.383–14.634), relative to the 40–49 years age bracket. In the asthma + COPD group, the OR of having asthma + COPD also increased with age: 60–69 years (3.720, 95% CI 2.304–6.008) and ≥70 years (6.399, 95% CI 3.824–10.709) relative to the 40–49 years age bracket. For household income, there were no associations among household incomes in the asthma group, whereas the low group (1.222, 95% CI 1.062–1.405) had a higher risk for COPD than the high group. Likewise, the low group (1.222, 95% CI 1.062–1.405) had a higher risk for asthma + COPD than the high group.

With respect to educational level, the association with asthma was higher among elementary school graduates (1.482, 95% CI 1.043–2.106) than among college graduates, whereas the association with COPD was higher among elementary school graduates (1.478, 95% CI 1.267–1.723), middle school graduates (1.303, 95% CI 1.107–1.534), and high school graduates (1.166, 95% CI 1.012–1.343) than among college graduates. In the asthma + COPD group, there was no statistically significant association. Based on occupation, machine fitting and simple labor (0.684, 95% CI 0.472–0.991) showed a lower association than being unemployed in the asthma + COPD group.

Among health behavior and disease‐related factors, overweight subjects (1.255, 95% CI 1.038–1.518) showed a higher association than those with normal weight in the asthma group, whereas underweight subjects (1.405, 95% CI 1.042–1.896) showed a high association with COPD, and overweight subjects (0.693, 95% CI 0.633–0.759) showed a low association with COPD. In the asthma + COPD group, underweight subjects (2.463, 95% CI 1.426–4.253) showed a high association with asthma + COPD. In the asthma group, there was no significant association with smoking status, whereas, in the COPD group, former smokers (1.546, 95% CI 1.336–1.790) and current smokers (2.336, 95% CI 2.029–2.689) showed a higher association than nonsmokers. A similar pattern was also found in the asthma + COPD group (former smokers: 1.728, 95% CI 1.095–2.726; current smokers: 2.145, 95% CI 1.320–3.484). With respect to alcohol consumption, individuals who consume any alcohol showed significantly lower associations with asthma and COPD than those who do not drink at all. In the asthma + COPD group, individuals who consume ≤1 drink per month showed a lower association. Individuals with a previous history of atopic dermatitis showed a higher association with asthma (3.175, 95% CI 1.792–5.624) and asthma + COPD (2.093, 95% CI 1.050–4.171), whereas individuals with a previous history of lung cancer showed a higher association with COPD (3.122, 95% CI 1.650–5.907).

## 4 DISCUSSION

The present study used reliable and nationally representative data to analyze the demographic characteristics, health behavior, disease‐related factors, and risk factors associated with respiratory diseases among 35 235 Korean adults aged ≥40 years.

In this study, women showed a high association with asthma than men. Such findings supported the results from a previous study reporting that 71% of adult asthma patients are women.^3^ Although the exact role of sex hormones in the regulation of asthma has not been identified, it has been reported that ovarian hormones exacerbate and testosterone alleviates asthmatic airway inflammation.^18^ Being overweight showed a higher association with asthma than being normal weight. This finding supports the results from a previous study reporting that obesity increased the risk of adulthood asthma by approximately 50% in both men and women.^19^ People with a previous history of atopic dermatitis showed a significantly high association with asthma (3.175, 95% CI 1.792–5.624), which appears to be associated with atopic march, a phenomenon involving atopic dermatitis progressing to allergic asthma and allergic rhinitis.^20^

Furthermore, women showed a lower association with COPD than men. Such findings support the fact that COPD is more frequent in men than women due to COPD being historically linked to smoking status and occupational exposure.^21^ This association increases with age, as compared with those aged 40–49 years, and in particular, the OR of COPD in individuals aged ≥70 years was approximately 12 times higher. These findings support results from previous studies indicating that the risk of COPD increases with age.^5^, ^12^ Concerning income quartiles, the association with COPD was lower in high‐income groups than that in the low‐income group. With respect to BMI, underweight individuals showed a high association with COPD, whereas individuals who are overweight actually showed a lower risk. The findings were similar to those of a study in 2011 reporting that low BMI is associated with COPD.^5^ Similar to previous studies, former and current smokers showed a higher association with COPD than nonsmokers.^22^ In particular, individuals with a previous history of lung cancer showed a higher association with COPD, which had also been reported in previous studies.^23^

Finally, individuals aged 60–69 and ≥70 years showed a higher association with asthma + COPD than those aged 40–49 years. These findings support the results from previous studies indicating that the prevalence of asthma + COPD increases significantly with age and that age is associated with asthma + COPD.^24^, ^25^ With respect to income quartiles and educational level, the risk factors of asthma + COPD showed largely similar characteristics as the risk factors of COPD, which supports previous studies reporting that many patients with COPD also have asthma + COPD and that patients with asthma + COPD share the same demographic characteristics and exhibit similar lung function test results as patients with COPD alone.^26^ The results also revealed that underweight individuals had a high association with asthma + COPD, which was contradictory to previous studies reporting that high BMI is associated with asthma + COPD.^27^ In the present study, the subjects were limited to adults aged ≥40 years. As a result, the association between asthma + COPD and COPD was even more pronounced. Consistent with COPD, former and current smokers showed a higher association with asthma + COPD than nonsmokers. Such findings could be considered similar to the results from previous studies showing no significant differences between the COPD and asthma + COPD groups in relation to smoking habits (former, current, and nonsmokers).^28^ Lastly, individuals with a previous history of atopic dermatitis showed a high association with asthma + COPD.^26^

Studies in the US have also used data from the KNHANES to investigate the status of respiratory diseases. The results revealed that the prevalence of asthma + COPD had increased from 0.96% to 1.05% between 2007 and 2012. The age‐standardized prevalence of asthma + COPD was higher among individuals with low socio‐economic status and previous history of myocardial infarction or stroke. Old age and smoking status showed even higher associations with the prevalence of asthma + COPD. Among the participants with COPD, a higher prevalence was correlated with being non‐Hispanic Black, being obese, and having a history of myocardial infarction or stroke. asthma + COPD is associated with the use of oxygen therapy for severe asthma and COPD. The participants with asthma + COPD showed a poorer FEV1 in the lung function test results than those with asthma or COPD alone.^29^ With respect to risk factors, old age, smoking status, and obesity showed similar results as the present study. Participants with a low socio‐economic status are highly likely to present severe COPD together with frequent and uncontrolled asthma for a long time.^9^

### 4.1 Strength and limitations

The present study had some limitations. First, because it was a cross‐sectional study using KNHANES data, caution should be taken when interpreting the causal relationships between the variables. In other words, whether the associated factors are independent causative factors or epiphenomenon factors cannot be known by their causal relationships. Second, asthma has a characteristic higher incidence at younger ages, but because the study population included only adults aged ≥40 years, the identification of factors may have been influenced by the fact that more COPD cases than asthma cases were included. Third, asthma was diagnosed by a doctor and thus may have been subject to underdiagnosis, overdiagnosis, or poor accessibility to health care services.^30^ On other hand, we included patients diagnosed based on the pulmonary function test (laboratory diagnostic criteria) for COPD. The diagnosis of asthma is made based on a physician's subjective judgment, the diagnosis may not be as objective as COPD. There is the additional confounder that the physician's diagnosis of asthma would have been based on a number of different factors in that age group, such as gender and smoking history. Lastly, KNHANES was not created for the purpose of investigating chronic airway diseases. Therefore, the total participation rate was limited, and only participants with vital capacity measurements were included. Because lung function was not measured in all individuals who participated in KNHANES, selection bias cannot be discarded. To overcome such shortcomings, large‐scale prospective cohort studies are needed to identify the associations of suspected risk factors and actual causes with outcomes of respiratory diseases.

Despite these limitations, the present study also had the following strengths. First, it used data from surveys conducted by a Korean government agency. The surveys were nationwide and were conducted on sample populations from the same region using the same questions during the same survey period. Because the surveys targeted the general population, the obtained data have high reliability. Second, this was the first study to analyze the risk factors of three different respiratory diseases over multiple years. While previous studies have analyzed the risk factors of respiratory diseases, no study has analyzed such factors over multiple years. Additionally, the present study compared the associations of asthma‐asthma + COPD and COPD‐asthma + COPD to investigate which risk factors would increase the likelihood of asthma or COPD progressing to asthma + COPD. Finally, the present study measured the ORs of each respiratory disease relative to a group of healthy controls to identify the risk factors that influence specific respiratory diseases.

This study analyzed the correlation between respiratory diseases and associated factors from 2008–2018. As a result of the global COVID‐19 pandemic, there may have been changes to the status of respiratory diseases since 2019. Subsequent studies can comparatively analyze the status of respiratory diseases prior to and after the advent of the COVID‐19 pandemic by examining respiratory diseases after 2019. Therefore, this study provides useful data shedding light on the characteristics of respiratory diseases in the Korean population prior to the advent of COVID‐19. In the future, studies should analyze respiratory diseases and their associated factors in Korea also using data since 2019.

## 5 CONCLUSION

The present study identified the associations between respiratory diseases and demographic factors, health behavior, and disease‐related factors. In particular, the independent risk factors influencing specific diseases were being a woman and having a previous history of atopic dermatitis for asthma; older age, being a current smoker, and having a previous history of lung cancer for COPD; and older age, being underweight, being a current smoker, and having a previous history of atopic dermatitis for asthma + COPD. Our findings could be used to developing policies to treatment of COVID‐19, respiratory diseases and the prevention of infectious diseases.

## CONFLICT OF INTEREST

The authors have no conflict of interests to declare.

## ETHICS STATEMENT

The KNHANES IV–VII were conducted by the Korea Centers for Disease Control and Prevention (KCDC) and written consent was obtained from all participants. All survey protocols were approved by the Institutional Review Board (IRB) of the KCDC (approval numbers: 2008‐04EXP‐01‐C, 2009‐01CON‐03‐2C, 2010‐02CON‐21‐C, 2011‐02CON‐06‐C, 2012‐01EXP‐01‐2C, 2013‐07CON‐03‐4C, 2013‐12EXP‐03‐5C, and 2018‐01‐03‐P‐A). The data from KNHANES is available on the ‘Korea National Health and Nutrition Examination Survey’ website (http://knhanes.cdc.go.kr). The present study was conducted after approval from the IRB (JASENG IRB File No. 2021‐05‐013).

## AUTHOR CONTRIBUTIONS

**Conceptualization:** Yoon Jae Won, Sook‐Hyun Lee, In‐Hyuk Ha. **Data curation:** Yoon Jae Won, Yu‐cheol Lim, In‐Hyuk Ha. **Formal analysis:** Yu‐cheol Lim, In‐Hyuk Ha. **Methodology:** Yoon Jae Won, Sook‐Hyun Lee, In‐Hyuk Ha. **Project administration:** Sook‐Hyun Lee, Beom‐Joon Lee, In‐Hyuk Ha. **Validation:** Yoon Jae Lee, Maurits Van den Noort, Beom‐Joon Lee. **Writing – original draft:** Yoon Jae Won, Sook‐Hyun Lee, In‐Hyuk Ha. **Writing – review & editing:** Yoon Jae Won, Sook‐Hyun Lee, Yu‐cheol Lim, Yoon Jae Lee, Maurits van den Noort, Beom‐Joon Lee, In‐Hyuk Ha.

## Supporting information


**Table S1.** Association of risk factors for asthma+COPD among Korean participants with asthma or COPD in the KNHANES 2008–2018

## Data Availability

The datasets generated and analyzed during the current study are available in the KNHANES repository (http://knhanes.cdc.go.kr). All data from KNHANES‐VI are coded and freely available.
