# Supplementary material for: Characteristics and difference of respiratory diseases in Korean adults aged ≥40 years: A cross‐sectional study
Source: Clin Respir J. 2022 Nov 15;17(1):29–39. doi: 10.1111/crj.13558 (PMC9829619; doi:10.1111/crj.13558)
Supplement: Supplementary file 1 — Table S1. Association of risk factors for asthma+COPD among Korean participants with asthma or COPD in the KNHANES 2008–2018 [file CRJ-17-29-s001.docx]

| **Supplementary Table 1. Association of risk factors for ASTHMA+COPD among Korean participants with asthma in the KNHANES 2008–2018**   \| Variables \| \| Asthma group  (n = 1,093) \| \| \|  \| COPD group  (n = 4,900) \| \| \| \| \| --- \| --- \| --- \| --- \| --- \| --- \| --- \| --- \| --- \| --- \| \| OR† \| 95% CI \| \|  \| OR† \| 95% CI \| \| \| \| Sex \| Men (ref.) \| 1.000 \|  \|  \|  \| 1.000 \|  \|  \| \| \| Women \| 0.234 \| 0.140 \| 0.392 \|  \| 2.235 \| 1.397 \| 3.577 \| \| \| Age (y) \| 40–49 (ref.) \| 1.000 \|  \|  \|  \| 1.000 \|  \|  \| \| \| 50–59 \| 2.012 \| 1.052 \| 3.850 \|  \| 0.589 \| 0.357 \| 0.974 \| \| \| 60–69 \| 4.141 \| 2.180 \| 7.870 \|  \| 0.594 \| 0.361 \| 0.976 \| \| \| ≥ 70 \| 6.479 \| 3.327 \| 12.615 \|  \| 0.589 \| 0.349 \| 0.992 \| \| \| Household income \| 1st quartile \| 1.240 \| 0.702 \| 2.189 \|  \| 1.262 \| 0.841 \| 1.893 \| \| \| 2nd quartile \| 1.091 \| 0.628 \| 1.895 \|  \| 0.967 \| 0.652 \| 1.434 \| \| \| 3rd quartile \| 1.203 \| 0.672 \| 2.155 \|  \| 1.010 \| 0.682 \| 1.496 \| \| \| 4th quartile (ref.) \| 1.000 \|  \|  \|  \| 1.000 \|  \|  \| \| \| Education \| Elementary (< 6 years) \| 1.018 \| 0.499 \| 2.080 \|  \| 1.030 \| 0.656 \| 1.617 \| \| \| Middle (7–9 years) \| 0.680 \| 0.316 \| 1.461 \|  \| 0.783 \| 0.467 \| 1.312 \| \| \| High (10–12 years) \| 0.985 \| 0.520 \| 1.869 \|  \| 0.755 \| 0.492 \| 1.159 \| \| \| College (> 12 years) (ref.) \| 1.000 \|  \|  \|  \| 1.000 \|  \|  \| \| \| Occupation \| Unemployed (ref.) \| 1.000 \|  \|  \|  \| 1.000 \|  \|  \| \| \| Professions \| 0.584 \| 0.045 \| 7.574 \|  \| 0.478 \| 0.115 \| 1.988 \| \| \| Office work \| 0.439 \| 0.188 \| 1.028 \|  \| 0.823 \| 0.429 \| 1.578 \| \| \| Sales and services \| 0.901 \| 0.337 \| 2.410 \|  \| 0.741 \| 0.343 \| 1.601 \| \| \| Agriculture, forestry and fishery \| 1.253 \| 0.599 \| 2.623 \|  \| 0.853 \| 0.468 \| 1.555 \| \| \| Machine fitting and simple labor \| 0.616 \| 0.365 \| 1.038 \|  \| 0.685 \| 0.465 \| 1.009 \| \| \| Others \| 1.295 \| 0.670 \| 2.503 \|  \| 0.771 \| 0.495 \| 1.201 \| \| \| BMI (kg/m2) ‡ \| Normal weight (ref.) \| 1.000 \|  \|  \|  \| 1.000 \|  \|  \| \| \| Underweight \| 5.272 \| 1.444 \| 19.246 \|  \| 1.804 \| 1.024 \| 3.177 \| \| \| Overweight \| 0.627 \| 0.445 \| 0.884 \|  \| 1.348 \| 1.035 \| 1.757 \| \| \| Smoking status \| Non-smoker (ref.) \| 1.000 \|  \|  \|  \| 1.000 \|  \|  \| \| \| Former \| 1.111 \| 0.623 \| 1.980 \|  \| 1.082 \| 0.680 \| 1.723 \| \| \| Current \| 1.649 \| 0.953 \| 2.853 \|  \| 0.928 \| 0.578 \| 1.490 \| \| \| Alcohol consumption \| None (ref.) \| 1.000 \|  \|  \|  \| 1.000 \|  \|  \| \| \| ≤ 1 drink/mo \| 0.920 \| 0.596 \| 1.420 \|  \| 0.793 \| 0.568 \| 1.107 \| \| \| 2 drinks/mo to 3 drinks/mo \| 1.213 \| 0.740 \| 1.988 \|  \| 0.780 \| 0.544 \| 1.117 \| \| \| ≥ 4 drinks/mo \| 1.579 \| 0.728 \| 3.425 \|  \| 0.834 \| 0.529 \| 1.315 \| \| \| Physical activity (Regular Walking) \| No (Rarely) (ref.) \| 1.000 \|  \|  \|  \| 1.000 \|  \|  \| \| \| Yes (Regularly) \| 1.006 \| 0.644 \| 1.572 \|  \| 1.012 \| 0.748 \| 1.369 \| \| \| Type of comorbidity \| Atopic dermatitis \| 0.715 \| 0.234 \| 2.191 \|  \| 1.936 \| 0.918 \| 4.082 \| \| \| Lung cancer \| 0.455 \| 0.060 \| 3.444 \|  \| 0.416 \| 0.053 \| 3.298 \| \| \| † Odds ratios with adjustments using logistic regression models adjusted for sex, age, household income, education, occupation, BMI, smoking status, alcohol consumption, physical activity, types of comorbidity; ‡ BMI was categorized into underweight (< 18.5 kg/m2), normal weight (18.5 ≤ BMI < 25 kg/m2), overweight (25 ≥ BMI kg/m2).  Abbreviations: **COPD**, chronic obstructive pulmonary disease; **OR**, adjusted odds ratio; **CI**, confidence interval; **BMI**, body mass index. \| \| \| \| \| \| \| \| \| |
| --- | --- | --- | --- | --- | --- | --- | --- | --- | --- | --- | --- | --- | --- | --- | --- | --- | --- | --- | --- | --- | --- | --- | --- | --- | --- | --- | --- | --- | --- | --- | --- | --- | --- | --- | --- | --- | --- | --- | --- | --- | --- | --- | --- | --- | --- | --- | --- | --- | --- | --- | --- | --- | --- | --- | --- | --- | --- | --- | --- | --- | --- | --- | --- | --- | --- | --- | --- | --- | --- | --- | --- | --- | --- | --- | --- | --- | --- | --- | --- | --- | --- | --- | --- | --- | --- | --- | --- | --- | --- | --- | --- | --- | --- | --- | --- | --- | --- | --- | --- | --- | --- | --- | --- | --- | --- | --- | --- | --- | --- | --- | --- | --- | --- | --- | --- | --- | --- | --- | --- | --- | --- | --- | --- | --- | --- | --- | --- | --- | --- | --- | --- | --- | --- | --- | --- | --- | --- | --- | --- | --- | --- | --- | --- | --- | --- | --- | --- | --- | --- | --- | --- | --- | --- | --- | --- | --- | --- | --- | --- | --- | --- | --- | --- | --- | --- | --- | --- | --- | --- | --- | --- | --- | --- | --- | --- | --- | --- | --- | --- | --- | --- | --- | --- | --- | --- | --- | --- | --- | --- | --- | --- | --- | --- | --- | --- | --- | --- | --- | --- | --- | --- | --- | --- | --- | --- | --- | --- | --- | --- | --- | --- | --- | --- | --- | --- | --- | --- | --- | --- | --- | --- | --- | --- | --- | --- | --- | --- | --- | --- | --- | --- | --- | --- | --- | --- | --- | --- | --- | --- | --- | --- | --- | --- | --- | --- | --- | --- | --- | --- | --- | --- | --- | --- | --- | --- | --- | --- | --- | --- | --- | --- | --- | --- | --- | --- | --- | --- | --- | --- | --- | --- | --- | --- | --- | --- | --- | --- | --- | --- | --- | --- | --- | --- | --- | --- | --- | --- | --- | --- | --- | --- | --- | --- | --- | --- | --- | --- | --- | --- | --- | --- | --- | --- | --- | --- | --- | --- | --- | --- | --- | --- | --- | --- | --- | --- | --- | --- | --- | --- | --- | --- | --- | --- | --- | --- | --- | --- | --- | --- | --- | --- | --- | --- | --- | --- | --- | --- | --- | --- | --- | --- | --- | --- | --- | --- | --- | --- | --- | --- | --- | --- | --- |
